# Postoperative Hemorrhage and Venous Thromboembolism in Patients with Pituitary Adenomas Under Acetylsalicylic Acid

**DOI:** 10.3390/jcm13237020

**Published:** 2024-11-21

**Authors:** Nikolay Tonchev, Anatoli Pinchuk, Claudia A. Dumitru, Klaus-Peter Stein, Belal Neyazi, I. Erol Sandalcioglu, Ali Rashidi

**Affiliations:** Department of Neurosurgery, Otto-von-Guericke-University Magdeburg, 39120 Magdeburg, Germany; nikolay.tonchev@med.ovgu.de (N.T.); anatoli.pinchuk@med.ovgu.de (A.P.); klaus-peter.stein@med.ovgu.de (K.-P.S.); belal.neyazi@med.ovgu.de (B.N.); erol.sandalcioglu@med.ovgu.de (I.E.S.)

**Keywords:** acetylsalicylic acid, pituitary adenoma, craniotomy, hemorrhage

## Abstract

**Background/Objectives:** Postoperative hemorrhages (POHs) after pituitary adenoma surgery can have devastating consequences for patients. Many patients take acetylsalicylic acid (ASA) for the primary or secondary prevention of cardiovascular or stroke events. However, the impact of continued low-dose ASA use on the risk of postoperative hemorrhage and the frequency of thromboembolic events after discontinuing ASA in these patients remain poorly understood. This study aims to investigate the potential interaction and correlation between low-dose ASA intake and two of the most common complications after neurosurgical surgery—acute postoperative hemorrhage and thromboembolism. **Methods:** A retrospective study involving 1862 patients who underwent brain tumor surgery over a decade at our neurosurgical institute examined the risk of postoperative hemorrhage and thromboembolic events. The study compared bleeding rates in patients with pituitary adenomas who received low-dose ASA medication to those who did not. Additionally, the study investigated the occurrence of venous thromboembolism (VTE) or arterial pulmonary embolisms (PEs) following surgery, as well as the impact of laboratory parameters, demographic characteristics and intraoperative factors. **Results:** A total of 108 patients underwent surgery for primary pituitary tumors between January 2008 and January 2018. Only six patients (5.6%) experienced POH. Among those with POH, just two (1.9%) required revision surgery due to neurological decline. Interestingly, none of the 13 patients (12%) taking ASA preoperatively suffered POH. No correlation was found between laboratory results, demographics and postoperative complications. The study also did not find an increase in VTE or PE events. **Conclusions:** In this analysis, the perioperative intake of low-dose ASA could not be associated with an increased rate of hemorrhagic complications following pituitary adenoma surgery. Low-dose ASA can be safely continued during brain tumor surgery in patients with a high cardiovascular and cerebrovascular risk.

## 1. Introduction

Pituitary adenomas, now more commonly referred to as pituitary neuroendocrine tumors (PitNETs) [1,2], are among the most frequent primary intracranial tumors [3,4]. The neurological symptoms range from visual disturbances in non-functioning adenomas [5] to high morbidity and mortality in secreting adenomas [6]. These symptoms often require surgical treatment. Pituitary adenomas account for approximately 10–15% of intracranial tumors [7], with the highest incidence occurring between the ages of 65 and 84 [8]. In the elderly population, the prevalence of pituitary adenomas reaches 15% [4,9,10]. Life expectancy in industrialized countries increased by about 30 years during the 20th century and continues to rise linearly [11,12,13]. In addition, perioperative morbidity and mortality rates are generally higher in elderly patients undergoing surgery [13,14,15,16,17]. Many elderly patients suffer from cardiovascular diseases [18] and ischemic strokes [19], making the use of antiplatelet agents increasingly important for the prophylaxis and secondary prevention of cardiovascular [20] and cerebrovascular diseases [7,21,22]. The same trend could be observed in the use of low-dose ASA, as one of the most commonly prescribed medications for primary and secondary prevention [23]. A meta-analysis on patients with brain tumors under therapeutic anticoagulation by Zwicker et al. found a greater than 3-fold increased risk of POH among the patients with glioma [24]. However, a later publication by Miller et al. reported no difference in major intracranial hemorrhage at one year in patients with metastatic brain tumors on antiplatelet medication [25]. Very few reports addressed the management of antiplatelet therapy in patients with intra- and parasellar tumors [7,26]. Enciu et al. compared the risk of postoperative bleeding in patients with low-dose ASA medication during endoscopic endonasal surgery for PA [7]. They reported that patients on ASA have a comparable risk of POH to patients without ASA, suggesting that antiplatelet medication could be safely continued in this patient group.

In most cases, the decision to discontinue antiplatelet medications prior to neurological surgery is made on a case-by-case basis and is based on the surgeon’s personal experience. The evaluation of perioperative risk has to take many side factors into account, such as comorbidities, the patient’s age, underlying oncological disease and much more, thus making it even more complex. Many reports depict an increased occurrence of peripheral venous thrombosis upon the surgical treatment of malignant brain tumors [27,28,29]. In high-risk groups of patients undergoing endoscopic transsphenoidal PA surgery, even lethal ischemic brain infarction has been reported [30]. In light of these findings, our analysis aims to clarify the perioperative risk of postoperative hemorrhage and venous thromboembolism with particular focus on predisposed patients undergoing both microscopic transsphenoidal and transcranial PA surgery. Additionally, our study takes into account the unique characteristics of the tumor, all while considering the influence of low-dose ASA medication. This research emphasizes the possible correlation between different risk factors for POH and aims to facilitate the decision-making process in perioperative ASA management in neurosurgery.

## 2. Materials and Methods

**Figure 1:**
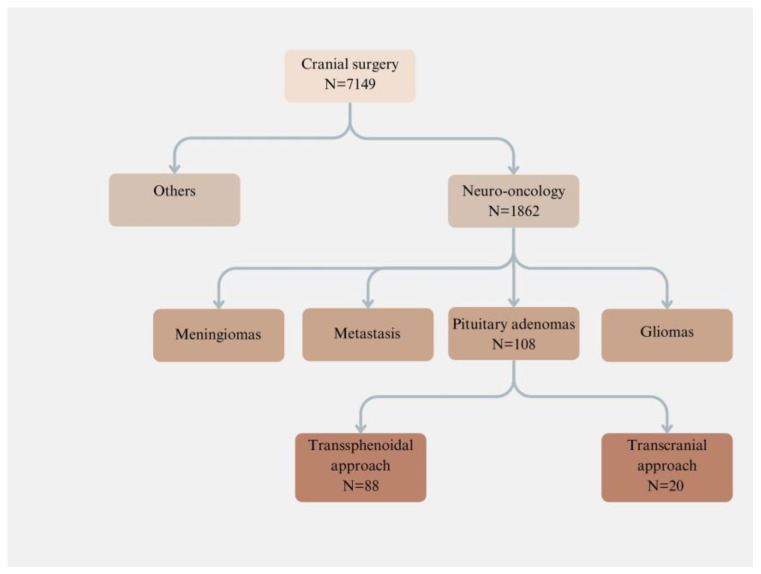
Types of tumors included in the study. We examined all brain tumor cases in our department over a 10-year period, identifying 108 patients with pituitary adenoma. Most of these patients (N = 88) were treated using the transnasal transsphenoidal approach. A transcranial approach was only chosen for a small proportion of patients (N = 20), mainly due to the considerable tumor extension in the suprasellar region.

A retrospective analysis was conducted on the medical records and radiological images of patients who underwent cranial surgery in our department between 2008 and 2018. This thorough review involved a total of 1862 tumor patients, with a specific focus on 108 patients who underwent transsphenoidal (N = 88) or transcranial (N = 20) surgery for pituitary adenomas, as illustrated in Figure 1.

Several clinical and patient-specific parameters were examined through comprehensive retrospective reviews of the medical records, including age, sex, blood type, body mass index (BMI), perioperative administration of low-dose ASA (100 mg/day), hypertension, diabetes, history of smoking, cardiovascular disease, renal disease, chronic inflammation, laboratory parameters, duration of hospital stay, type of surgical approach, duration of surgery, blood loss and postoperative complications.

Complications during hospitalization were classified according to the scheme proposed by Ibanez et al.—Grade I denoted non-life-threatening abnormalities within the typical postoperative course, manageable without invasive procedures; Grade II complications necessitated invasive interventions, including surgical, endoscopic or endovascular procedures; Grade III complications represented life-threatening adverse events requiring treatment in an intensive care unit and were further subdivided into IIIa for single organ dysfunction complications and IIIb for multiple organ dysfunction complications; Grade IV complications included deaths due to complications.

Preoperative contrast-enhanced MRI was used in the assessment of tumor volume, as well as for the evaluation of tumor extension. The extent of resection was evaluated according to operative protocols and postoperative MRI by two independent neurosurgeons. All patients received standardized postoperative 100 mg intravenous hydrocortisone substitution, followed by oral substitution according to our hospital’s protocol (postoperative day (POD) 1–2: 100 mg morning and 50 mg afternoon; POD 3–5: 75 mg morning and 50 mg afternoon; POD 6–7: 50 mg morning and 25 mg afternoon; POD 8: 40 mg morning and 25 mg afternoon; POD 9: 30 mg morning and 20 mg afternoon; POD 10: 20 mg morning and 10 mg afternoon).

Tumor characteristics, including exact histopathology, tumor size, tumor extension, recurrent surgery and degree of resection, were recorded precisely. Operative parameters such as blood loss during surgery, duration of surgery, extent of resection and other relevant characteristics were also registered. The pre- and postoperative Karnofsky Performance Status Scale (KPS) and the Glasgow Outcome Scale (GOS) after surgery provided a standardized assessment of patient status.

Exclusion criteria comprised patients below the age of 18, pregnant individuals and those receiving alternative antiplatelet agents like clopidogrel and/or anticoagulants (warfarin/DOACs).

To statistically assess the potential influence of ASA on postoperative bleeding, Fisher’s exact test was employed by categorizing the patients into two groups, as follows:

- No ASA impact—included patients without a history of ASA use and/or those who discontinued ASA intake (ceased ≥ 7 days before surgery).
- ASA impact—included patients who continued ASA intake (<7 days before surgery, with no cessation or partial cessation).

This classification was derived from the current European Society of Cardiology (ESC) and European Society of Anesthesiology (ESA) guidelines’ recommendations for patients undergoing neurosurgical operations [31].

Intracranial Hemorrhage Assessment:

All postoperative radiologic findings were reviewed for the presence of hemorrhage. Furthermore, two neurosurgeons independently verified the radiological images of all intracranial hemorrhages. Colleagues from the radiology department assisted with determining the blood volume.

Hemorrhages were systematically divided into the following categories:

- Hemorrhage within the tumor cavity;
- Intracerebral hemorrhage;
- Subarachnoid hemorrhage;
- Subdural hemorrhage.

Significant postoperative hemorrhages were exclusively recorded for patients exhibiting substantial neurologic symptoms and requiring surgical intervention due to space-occupying hemorrhages. Symptomatic neurologic deficits were precisely defined as focal neurologic deficit, headache, nausea or a change in cognitive functions.

### Statistical Analysis

Statistical analyses were performed with the SAS University Edition software package 9.4 (SAS Institute, Inc., Cary, NY, USA) and SPSS for Windows version 18.0 (SPSS, Inc., Chicago, IL, USA). Any *p*-value < 0.05 was indicative of a statistically significant result. Given the exploratory nature of the current analysis, it was intentionally assessed at the full significance level. For unadjusted analyses, Fisher’s exact test was employed for categorical variables, while the robust *t*-test (Satterthwaite) was applied to continuous variables. In cases where variables exhibited substantial deviation from the normal distribution, a logarithmic transformation was applied.

## 3. Results

During the complete observational period from 2008 to 2018, a total of 108 patients underwent pituitary adenoma surgery. Most of these patients (N = 88) underwent a microscopic endonasal transsphenoidal tumor resection. A smaller number of the cohort (N = 20) underwent transcranial tumor resection because of tumor extension in the suprasellar region. Almost one-fifth of the patients (18%) were treated for a recurrent tumor manifestation. Two patients were excluded from the statistical evaluation due to the absence of complete data records.

### 3.1. Incidence of Intracranial Hemorrhage

Six patients (5.6%) experienced postoperative hemorrhage. Among them, only two patients (1.9%) exhibited neurological symptoms or had hemorrhages of a space-occupying nature, necessitating revision surgery. Table 1 delineates the frequency of hemorrhage and the various categories thereof. A total of 14 patients (12.9%) had been taking low-dose ASA at the time of surgery, and of these, 13 (12.0%) were categorized in the group with ASA impact. Of note, patients with ASA impact did not have a significantly increased incidence of re-bleeding requiring surgery compared to the group without ASA intake (*p* = 0.987).

**Figure 2:**
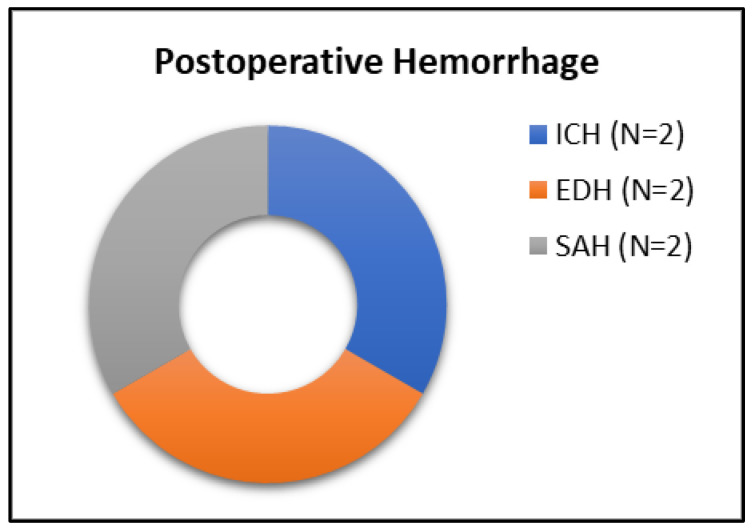
Two patients suffered intracerebral hemorrhage (ICH); two patients—epidural hemorrhage (EDH)—and two patients—subarachnoidal hemorrhage (SAH).

**Figure 3:**
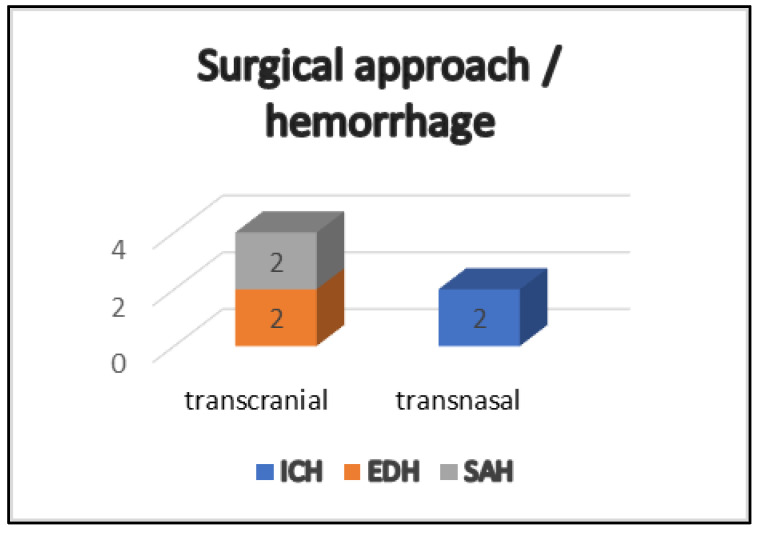
Four of the patients with postoperative hemorrhage were treated via the transcranial approach and only two via the transnasal one. The distribution of hemorrhage types is as follows: ICH (N = 2)—intracerebral hemorrhage; EDH (N = 2)—epidural hemorrhage; SAH (N = 2)—subarachnoidal hemorrhage.

The POHs exhibited variability, distinguishing between occurrences within the dural layers and those within the intraparenchymal space (Figure 2). Notably, only two patients (1.9%) presented with a hemorrhage in the resection cavity, specifically treated via a transnasal approach (Figure 3). However, these patients showed no neurological deterioration or deficits and were only diagnosed after regular postoperative radiologic imaging. The same number of patients (N = 2) presented with space-occupying epidural hematomas and were indicated for revision surgery. Hemorrhages in the subarachnoid space (N = 2) were also identified in two patients, who were operated upon using a transcranial approach. One of these two patients presented with a disturbed level of consciousness and anisocoria as signs of increased intracranial pressure on the seventh postoperative day and was indicated for VP-Shunt system implantation. Additional digital subtraction angiography (DSA) ruled out active arterial vessel bleeding. The second patient with postoperative SAH underwent a cardio-pulmonary resuscitation in the intraoperative period due to asystole, which was most probably also the reason for the neurological deterioration. Ten days after the surgery, he was discharged with a persistent visual imparement in his right eye. In total, none of the patients who experienced a hemorrhagic complication were under ASA medication.

### 3.2. Hemorrhage, Tumor Characteristics and Laboratory Parameters

No significant difference in the distribution of patients between the two groups (ASA impact and No ASA impact) was observed (*p* = 1.00).

Demographic factors and additional characteristics such as sex (*p* = 0.495), blood group (*p* = 0.915), smoking status (*p* = 0.494), age of the patient at the time of surgery (*p* = 0.643) and BMI (*p* = 0.932), as well as comorbidities including diabetes (*p* = 0.318), cardiovascular disease (*p* = 0.537), arterial hypertension (*p* = 0.934), dyslipoproteinemia (*p* = 0.775), renal disease (*p* = 0.842), liver disease (*p* = 0.748) and chronic inflammation (*p* = 0.680), demonstrated no significant impact on the risk of postoperative hemorrhage (Table 2).

The tumor characteristics, such as recurrent adenoma (*p* = 0.514), and surgical parameters such as the duration of surgery (*p* = 0.009) and intraoperative blood loss (*p* = 0.207) were evaluated as well (Table 3). The duration of surgery was clearly associated with a higher risk of POH in both patient groups. In particular, the GOS (*p* = 0.037) was significant in patients from both groups after hemorrhage (Table 4). The duration of hospitalization showed no significant trend in the comparison between patients with and without postoperative bleeding (*p* = 0.252).

Table 3 presents relevant preoperative laboratory parameters, especially coagulation factors. These data did not reveal any substantial influence on the risk of postoperative hemorrhage.

### 3.3. Intracranial Hemorrhage Relative to ASA Impact

Patients with and without ASA impact presented comparable distribution between sex (male/female), smoking history (*p* = 0.338) and with pre-existing conditions such as dyslipoproteinemia (*p* = 0.412), renal disease (*p* = 0.231), liver disease (*p* = 0.392), chronic inflammation (*p* = 0.983) and coagulopathy (*p* = 0.707). Contrary to this, patients with ASA impact presented statistically frequently with cardiovascular diseases (*p* = 0.001) and diabetes (*p* = 0.012) (Table 5).

### 3.4. VTE and ASA Intake

In the No ASA impact group, 2 out of 95 patients (2.1%) developed pulmonary artery embolism (PE), while none of the 13 patients in the ASA impact group experienced pulmonary artery embolism.

### 3.5. Complication According to Ibanez Classification

Table 6 illustrates the frequency of complications in both groups, utilizing the Ibanez classification for comparison. Surgical complications are classified as mild (Ibanez I—mostly transient neurological deficit), moderate (Ibanez II—mostly cerebrospinal fluid leaks and surgical site infections) and severe (Ibanez III—mostly hemorrhages or acute hydrocephalus; Ibanez IV—death). It is evident that there is no substantial difference between the two groups, whether with or without ASA impact. None of the patients under ASA developed severe medical or surgical complication. Notably, the percentage of patients (3.2%) developing VTE is comparable to that described in most previous publications. Other medical complications included urinary tract infection (1.1%) and lung failure, resulting in intubation or intensive care unit treatment (2.2%).

## 4. Discussion

The perioperative administration of antiplatelet agents as therapy or prophylaxis among neurosurgical patients remains a highly controversial topic. On one hand, an acute hemorrhagic event following neuro-oncological surgery can have a significant impact on the patients’ outcome [32,33]. On the other hand, thromboembolic complications can be clearly correlated with the cessation of ASA, as one of the most commonly prescribed antiplatelet agents [34,35]. Our findings demonstrate that patients with ASA impact were more vulnerable to complications due to the prevalence of multiple comorbidities, as illustrated in Table 5. The reported incidence of POH varies significantly among different studies, depending on the specific definition employed. In our cohort of pituitary adenoma patients, the occurrence of clinically relevant POH was 1.9%, which is within the range reported in the literature (0.8–6.9%) [36,37,38]. Therefore, analyzing the factors that contribute to these complications can be critical to improving patient outcomes.

The current study examined the prevalence and risk for hemorrhagic complications after pituitary adenoma surgery. There was no significant difference in the incidence of postoperative bleeding in patients taking ASA preoperatively. These findings are in line with similar observations in other neurosurgical studies focused on cranial and spine surgeries [39,40,41,42]. While some authors have reported an increased incidence of intracranial hemorrhage that is likely associated with ASA [43,44], other groups have found no significant impact of ASA on intracranial re-bleeding [7,45,46]. Focusing on patients with pituitary adenomas, a recent single-center study by Enciu et al. reported that a short-term discontinuation of low-dose ASA was not associated with an increased rate of postoperative bleeding in patients undergoing transnasal transsphenodial surgery. In their cohort, no patient from the ASA group underwent revision surgery due to POH, and only one patient without perioperative ASA medication underwent revision surgery for the treatment of POH with suprasellar extension [7]. However, these results are limited to endoscopic endonasal surgery, whereas in our analysis, we present the result from both microscopic and endoscopic transsphenoidal approaches for PA. In another retrospective study by Rahman et al. involving 451 patients who underwent brain tumor surgery, the analysis of those taking ASA compared to the control group revealed no significant difference regarding the occurrence of POH or thromboembolic systemic complications [45]. However, this study yielded limited results in terms of histological classification and tumor characteristics.

It should be mentioned that the cessation of ASA could potentially put patients with coronary artery disease and cerebrovascular or peripheral artery disease in a life-threatening situation. Several recent studies [47,48,49] revealed that perioperative ASA therapy was not associated with an increased risk of postoperative hemorrhage; therefore, it should be continued in patients at high risk of ischemic complications and low risk of bleeding in non-cardiac surgery. Graham et al. reported an absolute risk increase of 1.3% in patients with prior percutaneous coronary intervention (PCI) compared to 0.8% in the overall population [47]. In contrast to previous publications, our study focused not only on the hemorrhagic, but also on the thromboembolic complications. The incidence of venous thromboembolism (VTE) was comparable in both groups and was consistent with findings in other studies involving craniotomies. The aforementioned cerebrovascular accident has already been observed and reported in high-risk patients under ASA, while being surgically treated for pituitary adenoma [30]. However, there is considerable variability in reported data on VTE in brain tumor patients. For instance, one study indicated a 3.0% incidence of VTE in brain tumor patients undergoing craniotomy [27]. In our study, the frequency appeared to be 3.2%. Notably, two of those three patients were diagnosed with both DVT and PE and all three patients experienced several complications such as cerebrospinal fluid (CSF) leak and rhinorrhea, followed by continuous lumbar drainage treatment.

We could not identify any demographic parameters that had an impact on postoperative hemorrhagic complications. However, we still suggest that age and comorbidities play a significant role in the development of venous thromboembolism (VTE). This finding was recently described by Smith et al. in a single-center analysis focusing on venous thromboembolism in patients undergoing brain tumor surgery [50].

The results of our analysis on postoperative complication events may not be fully applicable to patients undergoing intra-axial brain tumor surgery, as PA is a mostly extra-arachnoid lesion with a typical transsphenoidal approach. However, a small part of our cohort was treated via a transcranial approach. Furthermore, our observation revealed that ASA is used for both primary and secondary prophylaxis in patients with various cardiovascular diseases, and is increasingly employed as an antiplatelet agent. This finding has been well observed and analyzed by other studies as well [23]. In the near future, even more elderly patients will require neurosurgical treatment, and the number of patients with an indication for antiplatelet therapy will also increase [51]. This makes the development of an appropriate evidence-based approach and risk assessment for these patients even more important.

## 5. Conclusions

Our study indicates that the continuation or short-term discontinuation of low-dose ASA therapy is not associated with an increase in symptomatic intracranial hemorrhage, requiring revision surgery. This result suggests that patients undergoing standard pituitary adenoma surgery can continue to take low-dose ASA for primary and secondary prophylaxis prior to surgery. An analysis of the patients’ demographics and other data revealed no significant differences between the two groups. However, it remains difficult to outline the exact perioperative risk on an individual basis.

The most important limitation of this study is its retrospective character. In addition, platelet function tests were not routinely performed prior to surgery, which limits the validity of the conclusions regarding ASA’s effect on hemostasis. Therefore, a further investigation including a Multiplate^®^ test is needed to detect probable non-responder patients. Although all surgeries in our department were conducted by certified specialists in the field of pituitary tumor surgery, variations in the surgical technique cannot be ruled out. Another limiting factor remains the low number of patients included in the ASA impact group.

By presenting our observational results, we hope to encourage a shift in the neurosurgical management of high-risk patients undergoing antiplatelet medication. Despite the clear findings from our analysis, further prospective studies and meta-analyses would be needed in order to establish secure treatment protocols for patients undergoing brain tumor surgery.

## Figures and Tables

**Table 1:** Classification of patients according to ASA impact and hemorrhagic complications leading to revision surgery.

				Hemorrhage with Operation	Hemorrhage Without Operation	No Hemorrhage	*p*-Value
				N (%)	N (%)	N (%)	
ASA impact			∑				
	Yes		13 (12.3)	0 (00.0)	0 (00.0)	13(13.0)	0.987
	No		93 (87.7)	2 (100.0)	4 (100.0)	87 (87.0)	
		∑ (%)	106	2	4	100	

**Table 2:** Patient distribution in accordance with demographic data, as well as comorbidities and tumor characteristics, and their correlation to hemorrhagic complications. None of these parameters showed significant influence on postoperative bleeding.

	Parameters		Hemorrhage with Operation	No Hemorrhage	*p*-Value
			N (%)	N (%)	
Demographic data					
	Sex	Female	0 (00.0)	51 (50.0)	0.495
		Male	2 (100.0)	51 (50.0)	
	Age [Mean ± SD]		63.0 ± 5.4	57.6 ± 14.6	0.643
	Blood group	A	1 (50.0)	39 (40.2)	0.915
		B	0 (0.0)	11 (11.3)	
		AB	0 (0.0)	9 (9.3)	
		O	1 (50.0)	38 (39.2)	
	Smoker	Yes	1 (50.5)	28 (28.3)	0.494
		No	1 (50.5)	71 (71.7)	
	BMI [Mean ± SD]		28.9 ± 0.5	29.3 ± 6.6	0.932
Comorbidities					
	Diabetes	Yes	1 (50.0)	17 (16.7)	0.318
		No	1 (50.0)	85 (83.3)	
	Hypertension	Yes	1 (50.0)	48 (47.1)	0.934
		No	1 (50.0)	54 (52.9)	
	Cardiovascular diseases	Yes	0 (0.0)	14 (13.7)	0.537
		No	2 (100.0)	88 (86.3)	
	Coagulopathy *	Yes	0 (0.0)	1 (1.0)	0.888
		No	2 (100.0)	101 (99.0)	
Tumor characteristics					
	Recurrence	Yes	0 (14.3)	18 (17.6)	0.514
		No	2 (14.3)	84 (82.4)	

BMI: body mass index; SD: standard deviation. * coagulopathy of insufficient clotting type.

**Table 3:** Laboratory parameters in relation to surgical outcome. None of the laboratory parameters showed a significant effect on postoperative hemorrhage risk. However, the duration of surgery showed a significant correlation with clinically relevant postoperative bleeding.

		Hemorrhage with Operation	No Hemorrhage	*p*-Value
		Mean ± SD	Mean ± SD	
Laboratory parameters				
	WBC count [Gpt/L]	9.1 ± 0.7	8.4 ± 2.9	0.390
	Platelet count [Gpt/L]	263.5 ± 51.6	275.9 ± 107.1	0.949
	Partial thromboplastin time [s]	28.0 ± 0.6	29.6 ± 6.5	0.632
	INR	1.0 ± 0.11	1.0 ± 0.21	0.907
	Thrombin time [s]	16.1 ± 0.64	16.6 ± 3.17	0.916
Intraoperative characteristics				
	Blood loss [mL]	450.0 ± 212	258.0 ± 247	0.207
	Duration of surgery [min]	376.5 ± 231.2	100.6 ± 57.3	**0.009**

**Bold** font represents statistically significant results (*p* < 0.05). WBC: white blood cell; Gpt: gigaparticle; INR: international normalized ratio; SD: standard deviation.

**Table 4:** Outcome parameters—hospital stay duration, together with KPS and GOS. Glasgow Outcome Scale showing a significant correlation with clinically relevant postoperative hemorrhage.

		Hemorrhage with Operation	No Hemorrhage	*p*-Value
		Mean ± SD	Mean ± SD	
Outcome patameters				
	Hospital stay [days]	25.0 ± 19.8	12.4 ± 6.3	0.252
	Karnofsky Performance Score	60.0 ± 28.3	74.3 ± 16.1	0.449
	Glasgow Outcome Scale	3.5 ± 0.7	4.6 ± 0.8	**0.037**

**Bold** font represents statistically significant results (*p* < 0.05). SD: standard deviation.

**Table 5:** Demographics, comorbidities, operative characteristics and outcome parameters of acetylsalicylic acid (ASA) groups. Patients in the ASA impact group were older than those in the No ASA impact group, had a poorer physical status (ASA score) and suffered more frequently from concomitant diseases such as diabetes and cardiovascular diseases.

	Parameters		ASA Preoperatively		*p*-Value
			No ASA Impact N 93 (%)	ASA Impact N 13 (%)	
Demographic data					
	Sex	Female	47 (50.5)	6 (46.2)	0.767
		Male	46 (49.5)	7 (53.8)	
	Age [Mean ± SD in years]		57.2 ± 14.8	61.6 ± 10.3	0.338
	BMI [Mean ± SD]		28.92 ± 6.45	30.45 ± 7.23	0.485
	ASA score	I	6 (6.5)	0 (0.0)	0.771
		II	66 (71.0)	9 (69.2)	
		III	21 (22.6)	4 (30.8)	
		IV	0 (0.0)	0 (0.0)	
	Smoker	Yes	28 (30.8)	2 (15.4)	0.338
		No	63 (69.2)	11 (84.6)	
Comorbidities					
	Diabetes	Yes	13 (14.0)	6 (46.2)	**0.012**
		No	80 (86.0)	7 (53.8)	
	Hypertension	Yes	52 (55.9)	9 (69.2)	0.089
		No	41 (44.1)	4 (30.8)	
	Cardiovascular diseases	Yes	7 (7.5)	6 (46.2)	**0.001**
		No	86 (92.5)	7 (53.8)	
	Coagulopathy *	Yes	1 (1.1)	0 (0.0)	0.707
		No	92 (98.9)	13 (100.0)	
	Chronic inflammation	Yes	7 (7.5)	1 (7.7)	0.983
		No	86 (92.5)	12 (92.3)	
Operative characteristics					
	Duration of operation [Mean ± SD in min]		109.3 ± 75.2	88.2 ± 39.7	0.405
	Blood loss [Mean ± SD in mL]		264.52 ± 246.36	221.15 ± 242.35	0.365
	Duration of hospital stay [Mean in days]		13.34	10.00	0.164
Outcome parameters					
	Karnofsky Performance Score [%]		73.44	76.15	0.960
	Glasgow Outcome Scale [1–5]		4.54	4.92	0.084

**Bold** font represents statistically significant results (*p* < 0.05). BMI: body mass index; SD: standard deviation. * coagulopathy of insufficient clotting type.

**Table 6:** Correlation between ASA impact and No ASA impact patients in both surgical and medical complication groups. There is no statistical difference in the occurrence of hemorrhagic and thromboembolic events between the two groups (ASA impact and No ASA impact).

According to Ibanez’ Classification:		ASA Impact N (%)	No ASA Impact N (%)	*p*-Value *
Surgical complication				
	None	12 (92.3)	72 (77.4)	0.894
	Ia/Ib	1 (7.7)	13 (14.0)	
	IIa/IIb	0 (0.0)	4 (4.3)	
	IIIa/IIIb	0 (0.0)	4 (4.3)	
	IVa/IVb	0 (0.0)	0 (0.0)	
	**∑**	13 (100.0)	93 (100.0)	
Medical complicaions				
	None	13 (100.0)	85 (91.4)	0.751
	Ia/Ib	0 (0.0)	1 (1.1)	
	IIa/IIb	0 (0.0)	3 (3.2)	
	IIIa/IIIb	0 (0.0)	2 (2.2)	
	IVa/IVb	0 (0.0)	2 (2.2)	
	∑	13 (100.0)	93 (100.0)	

* Examination with Fisher–Freeman–Halton Test. Complications are classified as mild (Ibanez I), moderate (Ibanez II) and severe (Ibanez III and IV).

## Data Availability

The datasets obtained and analyzed during the current study are available from the corresponding author on reasonable request.
